# Fire or Ice: Cryoablation as a Viable Alternative to Radiofrequency Ablation for Renal Artery Denervation?∗

**DOI:** 10.1016/j.jacbts.2021.10.019

**Published:** 2022-02-28

**Authors:** Saif Anwaruddin, Deepak L. Bhatt

**Affiliations:** a Department of Cardiovascular Medicine, St. Vincent Hospital, Worcester, Massachusetts, USA; b Division of Cardiovascular Medicine, Brigham and Women’s Hospital and Harvard Medical School, Boston, Massachusetts, USA

**Keywords:** cryoablation, hypertension, radiofrequency ablation, renal artery denervation

**Figure:**
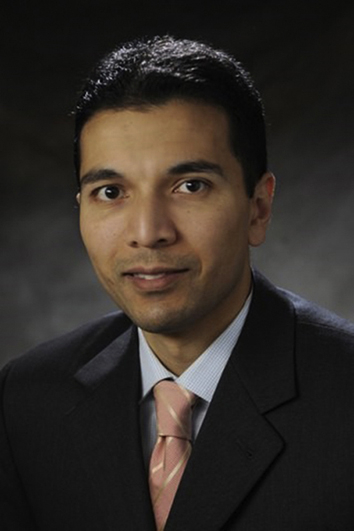

Some say the world will end in fire,

Some say in ice.

From what I’ve tasted of desire

I hold with those who favor fire.

But if it had to perish twice,

I think I know enough of hate

To say that for destruction ice

Is also great

And would suffice.

—Robert Frost (1)

Arterial hypertension is a highly prevalent and important modifiable risk factor in the prevention of subsequent cardiovascular disease. Despite effective pharmacologic treatments, inadequate blood pressure (BP) control remains an important issue. Resistant hypertension (RH) increases the risk of both cardiac and noncardiac target organ damage. Renal artery denervation (RDN) has the potential to be an important therapy in the management of RH. Although the initial RDN experience demonstrated the promise of RDN for RH, the pivotal SYMPLICITY-HTN-3 (Renal Denervation in Patients With Uncontrolled Hypertension) trial did not demonstrate superiority of RDN compared with sham in reduction of systolic blood pressure (SBP) (2). There were many postulated reasons for these findings, including operator experience, issues with the medical therapy regimen, and incomplete RDN. Design of more advanced catheters and modification of the treatment approach have moved the field of RDN forward with positive results in the SPYRAL HTN-OFF MED (Global Clinical Study of Renal Denervation With the Symplicity Spyral ™ Multi-Electrode Renal Denervation System in Patients With Uncontrolled Hypertension in the Absence of Antihypertensive Medications) study, with modest improvements in BP and a heightened enthusiasm (3). However, as the prior experience with RDN has taught us, there is always room for improvement.

The use of radiofrequency ablation (RFA) has been a mainstay of RDN catheter design and has been effective in reducing neurofilaments in animal models and effective in SBP reduction in patients. Although RFA RDN may be useful for BP reduction, there are reports of nonresponders following the procedure. Alternative methods of RDN are actively being explored, including endovascular ultrasound RDN as a promising option.

Cryoenergy as a potential method for RDN takes its cue from catheter ablation of atrial arrhythmias as an effective and less painful technique. One of the limitations of cryoablation for atrial arrhythmias is damage to structures in proximity, notably the phrenic nerve. More extensive nerve damage in the renal artery presumably may be advantageous in achieving more complete denervation and SBP reduction with this technique. The concept of cryoenergy for RDN (cryo-RDN) has previously been demonstrated to be effective at elimination of neurofilament bundles in ablated segments in animal models without damage to the endothelium or evidence for vascular injury or thrombosis, and, in patients who were considered nonresponders after RFA RDN, a cryoablation catheter was demonstrated to be effective in reducing both office-based BP and 24-hour ambulatory BP (4).

In setting the stage for the use of cryo-RDN for hypertension, preclinical data from pigs randomized to cryo-RDN using a balloon technique to deliver the cryoablation versus renal angiography demonstrated safety of the procedure, reduction in BP, postablation changes to neural tissue, and reduction of norepinephrine in the renal tissue (5).

In this issue of *JACC: Basic to Translational Science*, the same group led by Ji et al (6) present a proof of concept using their own dedicated cryoablation balloon catheter system using liquid nitrogen to perform RDN. The authors should be congratulated not only for their efforts in presenting novel data on cryo-RDN, but also for the demonstrated innovation in designing their own catheter system for this purpose. The study has both a preclinical and clinical component evaluating the safety and efficacy of this novel balloon catheter cryo-RDN system. The apparent advantage of their balloon catheter system design resulted in >80% circumferential injury and subsequent nerve damage in all histologic samples out to 6 months. Emphasizing complete and sustained nerve damage is important for the effectiveness of the procedure, and the lack thereof was likely a major contributing factor in the absence of significant benefit in the SYMPLICITY HTN-3 trial. The nerve damage was sustained as tyrosine hydroxylase staining remained low even out to 6 months, suggesting a durable response. Histologic evaluation did not demonstrate damage to surrounding structures or organs. In addition to the structural changes noted, there was a concomitant physiologic reduction in norepinephrine concentrations both in the kidneys and the systemic circulation.

The clinical correlation to this evaluated the cryo-RDN balloon in 6 patients with RH. Importantly, the proof of concept first in human demonstration was performed without issues in these patients and met its primary safety endpoint with no adverse events reported either clinically or angiographically. Albeit from a small group of patients, the largest reduction in SBP for both office-based BP and 24-hour ambulatory BP following cryo-RDN was at 6 months after the procedure. Interestingly, sustained and continued SBP improvement over time seems to correlate well with the histologic and physiologic findings from the animal models. It remains to be seen whether these clinical results will be reproducible in a large randomized clinical cohort of patients with appropriate sham controls.

Although this is a proof-of-concept study with few patients, understanding the mechanism and histologic findings in the context of clinical findings is important. In RFA RDN, animal models support reinnervation occurring as early as 5.5 months after the procedure and normal responses to electrical stimulation by 11 months, which has been observed previously after denervation (7). Although certainly not a direct comparison, the histologic findings after cryo-RDN and lack of reinnervation at 6 months should lead one to ponder whether the further reduction in SBP observed at 6 months is influenced by the sustained loss of neural tissue observed, although physiologic nerve stimulation was not performed. It is certainly plausible from a mechanistic standpoint that cryo-RDN with the specially designed balloon may be effective in more complete denervation leading to less re-innervation. Whether this translates to more sustained and better SBP reduction than RFA RDN remains to be seen. The authors do mention that SBP reductions are comparable with those seen in other contemporary studies, although large, randomized sham-controlled trials of cryo-RDN are needed.

This study is important in that it introduces 2 new variables into the RDN equation. First, the use of a balloon catheter that appears to make consistent circumferential contact to the vessel wall with high rates of tissue injury will likely help to make the procedure faster and potentially achieve more effective ablation. Second, the use of cryoenergy may be more effective at denervation with a more sustained result—only time and more data will tell. In the context of this study, how do we know whether the observed benefits are because of the balloon versus the type of energy? We cannot definitively say, however, the prior study of cryo-RDN in patients previously having failed RFA RDN using standard arrhythmia cryoablation catheters lends credibility to the idea that cryo-RDN itself may be an effective strategy and may act synergistically with the specially designed balloon catheter. Again, larger-scale clinical trials conducted in a randomized fashion with sham controls may provide definitive evidence in favor of cryo-RDN. At some point down the road, this will need to be followed by head-to-head clinical studies evaluating cryo-RDN and RFA RDN to examine comparative efficacy, safety, cost-effectiveness, and other important endpoints. Not unlike Robert Frost who contemplated the end of the world either in a fiery or a frigid finish, there may be different avenues to achieve successful and effective renal denervation and we must remain open minded (albeit in a more positive context) about the possibilities to accomplish the task at hand.

## Funding Support and Author Disclosures

Dr Anwaruddin is a consultant with Medtronic and Edwards Lifesciences; a proctor for Medtronic and Edwards Lifesciences; a member of the Advisory Board for Medtronic and OpSens; a member of the Steering Committee for Boston Scientific; and holds equity in East End Medical. Dr Bhatt is a member of the Advisory Board for Boehringer Ingelheim, Cardax, CellProthera, Cereno Scientific, Elsevier Practice Update Cardiology, Janssen, Level Ex, Medscape Cardiology, MyoKardia, NirvaMed, Novo Nordisk, PhaseBio, PLx Pharma, Regado Biosciences, Stasys; a member of the Board of Directors for Boston VA Research Institute, Society of Cardiovascular Patient Care, and TobeSoft; the Inaugural Chair of the American Heart Association Quality Oversight Committee; a member of the Data Monitoring Committees for Baim Institute for Clinical Research (formerly Harvard Clinical Research Institute, for the PORTICO trial, funded by St. Jude Medical, now Abbott), Boston Scientific (Chair, PEITHO trial), Cleveland Clinic (including for the ExCEED trial, funded by Edwards), Contego Medical (Chair, PERFORMANCE 2), Duke Clinical Research Institute, Mayo Clinic, Mount Sinai School of Medicine (for the ENVISAGE trial, funded by Daiichi Sankyo), Novartis, Population Health Research Institute; and has received honoraria from the American College of Cardiology (Senior Associate Editor, Clinical Trials and News, ACC.org; Chair, ACC Accreditation Oversight Committee), Arnold and Porter law firm (work related to Sanofi/Bristol-Myers Squibb clopidogrel litigation), Baim Institute for Clinical Research (formerly Harvard Clinical Research Institute; is a member of the RE-DUAL PCI clinical trial steering committee funded by Boehringer Ingelheim, the AEGIS-II executive committee funded by CSL Behring), Belvoir Publications (Editor in Chief, *Harvard Heart Letter*), Canadian Medical and Surgical Knowledge Translation Research Group (clinical trial steering committees), Cowen and Company, Duke Clinical Research Institute (clinical trial steering committees, including for the PRONOUNCE trial, funded by Ferring Pharmaceuticals), HMP Global (Editor in Chief, *Journal of Invasive Cardiology*), *Journal of the American College of Cardiology* (Guest Editor; Associate Editor), K2P (Co-Chair, interdisciplinary curriculum), Level Ex, Medtelligence/ReachMD (CME steering committees), MJH Life Sciences, Piper Sandler, Population Health Research Institute (for the COMPASS operations committee, publications committee, steering committee, and USA national co-leader, funded by Bayer), Slack Publications (Chief Medical Editor, *Cardiology Today’s Intervention*), Society of Cardiovascular Patient Care (Secretary/Treasurer), WebMD (CME steering committees); Other: *Clinical Cardiology* (Deputy Editor), NCDR-ACTION Registry Steering Committee (Chair), VA CART Research and Publications Committee (Chair); Research Funding: Abbott, Afimmune, Amarin, Amgen, AstraZeneca, Bayer, Boehringer Ingelheim, Bristol-Myers Squibb, Cardax, CellProthera, Cereno Scientific, Chiesi, CSL Behring, Eisai, Ethicon, Faraday Pharmaceuticals, Ferring Pharmaceuticals, Forest Laboratories, Fractyl, Garmin, HLS Therapeutics, Idorsia, Ironwood, Ischemix, Janssen, Lexicon, Lilly, Medtronic, MyoKardia, NirvaMed, Novartis, Novo Nordisk, Owkin, Pfizer, PhaseBio, PLx Pharma, Regeneron, Roche, Sanofi, Stasys, Synaptic, The Medicines Company, 89Bio; Royalties: Elsevier (Editor, *Cardiovascular Intervention: A Companion to Braunwald’s Heart Disease*); Site Co-Investigator: Abbott, Biotronik, Boston Scientific, CSI, St. Jude Medical (now Abbott), Philips, Svelte; Trustee: American College of Cardiology; and unfunded research from FlowCo, Merck, and Takeda.
